# Effective Strategies for Global Health Research, Training and Clinical Care: A Narrative Review

**DOI:** 10.5539/gjhs.v7n2p119

**Published:** 2014-09-28

**Authors:** Rebekah J. Walker, Jennifer A. Campbell, Leonard E. Egede

**Affiliations:** 1 Center for Health Disparities Research, Medical University of South Carolina, Charleston, SC, United States; 2 Health Equity and Rural Outreach Innovation Center, Charleston VA COIN, Ralph H. Johnson VA Medical Center, Charleston, SC, United States; 3 Division of General Internal Medicine and Geriatrics, Department of Medicine, Medical University of South Carolina, Charleston, SC, United States

**Keywords:** clinical care, global health, program development, research, training

## Abstract

The purpose of this narrative review was to synthesize the evidence on effective strategies for global health research, training and clinical care in order to identify common structures that have been used to guide program development. A Medline search from 2001 to 2011 produced 951 articles, which were reviewed and categorized. Thirty articles met criteria to be included in this review. Eleven articles discussed recommendations for research, 8 discussed training and 11 discussed clinical care. Global health program development should be completed within the framework of a larger institutional commitment or partnership. Support from leadership in the university or NGO, and an engaged local community are both integral to success and sustainability of efforts. It is also important for program development to engage local partners from the onset, jointly exploring issues and developing goals and objectives. Evaluation is a recommended way to determine if goals are being met, and should include considerations of sustainability, partnership building, and capacity. Global health research programs should consider details regarding the research process, context of research, partnerships, and community relationships. Training for global health should involve mentorship, pre-departure preparation of students, and elements developed to increase impact. Clinical care programs should focus on collaboration, sustainability, meeting local needs, and appropriate process considerations.

## 1. Introduction

The interest in global health has increased dramatically in the past decade. More medical students are taking international clinical and research opportunities (Smith & Weaver, 2006; Drain et al., 2007; Scarlett et al., 2011), and global health courses doubled in a three year time span (Scarlett et al., 2011). Recognition that global health inequities arise from the conditions in which people live, has caused global health programs to consider the social, economic, political, cultural and environmental determinants of health (World Health Organization, 2011). Students with international health experience are more likely to care for impoverished patients in the future and are more likely to express interest in volunteering, humanitarian efforts and serving multicultural and under-served patients (Smith & Weaver, 2006; Gupta, Wells, Horwitz, Bia, & Barry, 1999; Godkin, & Savageau, 2003; Thompson, Huntington, Hunt, Pinksy, & Brodie, 2003). Additionally, international health electives have been shown to increase cultural competence, compassion for patients, communication skills, and awareness of resource use (Mutchnick, Moyer, & Stern, 2003).

Concerns exist, however, regarding how best to structure and implement global health programs. The literature draws attention to the need to consider unintended consequences and ethical concerns (Green, Green, Scandlyn, & Kestler, 2009). Unfortunately, many physicians completing medical education lack specific training to impact health inequities, which include proficiency as broad as public health, health advocacy, program development, economics, ethics, and research skills (Garner, Kale, Dickson, Dans, & Salinas, 1998). Additionally, clinical care in resource-constrained settings differs dramatically from resource-rich settings (Furin et al., 2006). While there is a great deal of published literature on ethics, guidelines and models for global health, no systematic review on effective strategies exists. Disease specific reviews are more common than reviews on effective strategies for research, training and clinical care across diseases and cultures.

The purpose of this systematic review is to synthesize the evidence on effective strategies for global health research, training and clinical care in order to provide common structures that have been used to guide program development. Articles included in this review focused on ethics, guidelines and models for global health as defined by the Institute of Medicine where “health problems, issues, and concerns that transcend national boundaries, may best be addressed by cooperative actions” (Institute of Medicine, 1997). Public and private efforts, theoretical and practical frameworks, and existing and new programs were also included.

## 2. Materials and Methods

Medline was searched for articles published from January 2001 through December 2011 using a reproducible strategy. Three broad searches were conducted, the first using the term *global health*, the second using the terms *global health* and *training*, and the third using the terms *global health* and *academic medicine*. Duplicates were removed, producing 951 citations. Titles and abstracts were reviewed to determine whether articles were obviously ineligible, for instance focused on a review of global health efforts on a specific disease. Abstracts were also reviewed to determine whether articles addressed training, education, medical care, or collaboration in global health. This produced 204 articles for full review.

Full articles were read by two reviewers and categorized into project guidelines/ethics (103 articles), program models (57 articles), and training projects (44 articles). Eligibility of articles was determined by two reviewers and categorized into one of three overarching categories of recommendations: research (11 articles), training (8 articles) and clinical care (11 articles). This produced 30 articles for inclusion in the manuscript.

The context of each article and recommendations made by the authors were summarized. A table was compiled for recommendations from each of the main overarching categories: research, training and clinical care. After all articles in a category were read and summarized, recommendations were reviewed for themes within the three overarching categories. Two reviewers categorized each recommendation into a theme and compiled implications for each category and overall. Six tables were produced to display summarized recommendations and themes present in each article.

## 3. Results

Eleven articles were reviewed regarding effective strategies for research (Table 1). Four focused on a specific study (Treloar & Graham, 2003; Rojas, Lozano, & Rojas, 2007; Simon, Mosavel, & van Stade, 2005; Mosavel, Simon, van Stade, & Buchbinder, 2005), four focused on frameworks for research or evaluation of research (Huynen, Martens, & Hilderink, 2005; Lavery et al., 2010; Costello, & Zumla, 2000; Tugwell et al., 2006), and three largely addressed ethical considerations (Tan-Torres, 1999; Nishtar, 2004; Macrae, 2007). Six of the articles gave recommendations regarding process considerations (Treloar & Graham, 2003; Simon et al., 2007; Lavery et al., 2010; Tugwell et al., 2006; Nishtar, 2004; Macrae, 2007), seven addressed appropriate research context considerations (Simon et al., 2007; Mosavel et al., 2005; Huynen et al., 2005; Costello, & Zumla, 2000; Tugwell et al., 2006; Nishtar, 2004; Macrae, 2007), five addressed partnerships in research (Rojas et al., 2007; Huynen et al., 2005; Costello, & Zumla, 2000; Tan-Torres, 1999; Nishtar, 2004), and five gave recommendations on the importance of community relationships (Simon et al., 2007; Mosavel et al., 2005; Lavery et al., 2010; Costello, & Zumla, 2000; Tugwell et al., 2006; Tan-Torres, 1999), see Table 2.

**Table 1:** Summary of articles focused on research

Author/Year	Context/Summary	Recommendations
Treloar & Graham, 2003	Use of two international projects to discuss multidisciplinary cross-national qualitative studies.	Appropriate Funding: · Need funding for qualitative phase separate from quantitative phase · Lack of face-to-face meetings made working on new technique difficult and further alienated those unaccustomed to qualitative research · Needed for publications Need for Standardization: · Discrepancies in ethical standards between medical and social scientists · Differences in protocol development between investigators · Lack of training led to inexperienced interviewers and interpretation problems Flexibility: · Consider stepwise linking of centers or co-opting of social scientists to ensure appropriate support and resources · Respond to each center’s unique needs or concerns
Rojas et al., 2007	Example model of comprehensive approach to address health related international collaborative research in resource poor countries using infant health in Columbia, South America as an example.	Strengthen Research Capacity: · Support building and retaining research capacity in resource-poor nations through promoting models of collaborative research that are not paternalistic · Promote development and sustainability of capacity by using available capacity in resource-poor nations · Discuss research design and ethical issues in separate meetings from planning meetings to build expertise in design and conduct of high quality clinical research · Assign bulk of money to organizations in resource-poor country to promote fund implementation and coordination · Place emphasis on training future leaders that address relevant needs Promote Collaborative Process: · Assign specific roles to each partner · Hold meetings to describe projects and receive feedback on interest and feasibility · Include equity in setting research priorities
Simon et al., 2007	Critical examination of concept of relevance and community engagement in community-based health research using recent theory, literature and case study from South Africa.	Keep Research Design Open and Flexible: · Provide opportunities for comments, discussion of needs and concerns and recommendation of changes · Gain input, support, and cooperation from community · Do not formulate research question or hypothesis before speaking to community · Take into account how community need and crisis affects ethics of conducting research Invite Community Member Participation: · Provide opportunities for community to get to know researchers and ask questions, build rapport and mutual trust · Involve community as staff to provide advice and divide labor · Train community members to conduct and analyze results Opportunity for Continued Discussion: · Ethical responsibility to address differences and disconnections · Play a role in promoting dialogue, mutual respect and possible collaboration between different sectors of community
Mosavel et al., 2005	Discussion of community based participatory research as means to negotiate mutual agenda between community and researchers in South Africa.	Build Relationships: · Introduce team to stakeholders and establish dialogue and credibility · Invite local critique and feedback · Work closely with and learn from local researchers and stakeholders · Involve non-mainstream community members · Importance of strong communication skills Commit to Social, Political and Economic Context: · Develop interventions that have realistic expectations on outcome and based on what people can do · Work alongside policy rather than in opposition to it · Address tension between research and service delivery · Remain aware of ethical challenges
Huynen et al., 2005	Conceptual framework for health impacts of globalization to provide insights into how to organize complexity and give a meaningful contribution through research.	Recognize Complexity: · Multiple links between *Global Health* · Consider scenario analysis to explore possible pathways of impact · Contextual level factors influence distal factors such as policy, development, trade, and social interactions Use Interdisciplinary Approach: · Necessary to draw on multiple fields such as medicine, epidemiology, sociology, political science, education, and economics · Population health is outcome of social-cultural, economic and institutional determinants
Lavery et al., 2010	Describe framework for broader discussions of community engagement developed as a guide for a collaborative study in Mexico.	Build Capacity: · Select sites with capacity to be active participants · Initiate activities early to build knowledge and engage effectively · Identify and mobilize community skill sets and knowledge · Maximize leadership and ownership within community Provide Appropriate Information: · Ensure purpose and goals of research are clear · Give adequate information to permit reasonable judgment about research · Consider balance between informed consent and collective community authorization Establish Relationships: · Relationships of trust with key individuals and groups · Seek to understand perceptions and attitudes, not just measure them · Respect dissenting opinions and will of community · Grapple with questions and obligations to community
Costello & Zumla, 2000	Discussion on research partnerships in developing countries and principles behind research models.	Equal Research Partnerships: · Mutual trust and shared decision making · Nurture partnerships and developing institutions Develop Capacity: · Attention to ownership, sustainability and development of capacity · Build local academic infrastructure through line management of funds in local academic institutions, advice and technical support Emphasize Translation to Policy and Practice: · Provide dissemination to national or regional journals, not only international Incorporate policy considerations at start of project
Tugwell et al., 2006	Development of framework to assess capacity of low and middle-income countries for equity oriented research.	Knowledge Sharing: · Mechanisms to increase translation and impact on health equity · Packaging and implementation of research results Long-Term Capacity: · Need ability to manage knowledge and interpret results in political and social context and facilitate decision-making · Increase link to equity-focused research and alignment of research with health priorities · Avoid ‘project mentality’ and integrate operations of research into health programs · Lack of funding to strengthen capacity
Tan-Torres Edejer, 1999	Discussion of ethics involved in research especially as it pertains to collaborating with developing countries.	Collaboration: · Priority setting together that accommodates needs of developing country · Build mutual trust, share information and develop national research networks · Disseminate and apply results · Judge success on both results and working relationship Use Long Term Perspective: · Share responsibility and profits equitably · Involve local expertise in efforts to build national capacity
Nishtar, 2004	Key ethical and procedural issues of public-private partnerships in global health research.	Leverage Strengths of Partners: · Strategic partnership to provide resources, technical expertise or outreach to meet public good · Require well-defined governance to establish responsibility on part of all partners · Minimize skewed power relationships and promote mutually synergistic partnerships Benefit to Society: · Keep missions separate to focus on benefit to society rather than benefit to partners · Address conflict of interest and negative impacts of work such as fragmentation of health system · Focus on sustainability and delivery of efficient, effective and equitable services · Avoid duplication and complement national health priorities Transparency: · Require accountability to shared risks and process oriented action · Develop transparent policies and procedural frameworks · Assist in development of guidelines that are flexible
Macrae, 2007	Historical context of clinical trial ethics and international ethical guidelines.	Consent: · Ensure capacity for consent, voluntary consent and sufficient information given · Treat consent as process, not an event, providing time for questions · Use appropriate language and cultural sensitivity Intended Benefit: · Research with vulnerable populations should be intended to benefit group and provide therapeutic results · Research should pose minimal risk

**Table 2:** Recommendation themes for research

Author/Year	Process Considerations	Research Context	Partnerships	Community Relationships
Treloar & Graham, 2003	x			
Rojas et al., 2007			x	
Simon et al., 2007	x	x		x
Mosavel et al., 2005		x		x
Huynen et al., 2005		x	x	
Lavery et al., 2010	x			x
Costello & Zumla, 2000		x	x	x
Tugwell et al., 2006	x	x		x
Tan-Torres Edejar, 1999			x	x
Nishtar, 2004	x	x	x	
Macrae, 2007	x	x		

Eight articles were reviewed regarding effective strategies for training (Table 3). One article focused on training within the host country (Leonard, 2002), four articles were based on lessons learned from multi-institutional experiences (Rojas et al., 2007; Spiegal et al., 2011; Elit et al., 2011; Provenzano et al., 2010)., and three articles were based on development of new training programs or guidelines for development of programs (Furin et al., 2006; Redwood-Campbell et al., 2011; Crump, & Sugarman, 2010). Six of the eight articles gave recommendations for preparation of students, four commented on the importance of long term commitments, four noted aspects used to increase impact, and three stated the need for mentorship (Table 4).

**Table 3:** Summary of articles focused on training

Author/Year	Context/Summary	Recommendations
Leonard, 2002	Comparison between government and NGO health care in African countries. Recommendations on training of government health care systems.	Allow Flexibility: · Increase self-regulation by decentralized control over staffing · Delegation of authority at local level with national regulation Invest in Organizational Quality: · Increase reputation of staff regarding medical effort · Stress importance of completeness of examination, appropriate use of laboratory tests, attentiveness to the client, and health education
Spiegel et al., 2004	Discussion of partnership between University of British Columbia and network of Ecuadorian universities to develop a training program to improve environmental health conditions of vulnerable populations in Ecuador.	Institutional Commitment: · Necessary for success and sustainability of initiatives · Consolidation of support and continued interaction with leadership · Reinforce regional networks rather than only bilateral relationships · Ensure national collaboration through coordinated effort · Address issues of operationalizing commitment and maintaining continuity · Development of teaching materials in collaborative manner led to broader partnerships · Strengthen human resources and institutional capabilities through efforts · Building a strong multi-disciplinary team allowed for wide range of expertise to establish networks · Engage community members to intensify communication and collaboration Clearly Articulated Vision: · Clear vision allowed for emphasis to remain on application of knowledge to achieve impact · Outcome targets could be set with intended outcomes and intended impacts based on vision
Furin et al., 2006	Description of graduate *Med Educ* program in health equity developed at Bringham and Women’s Hospital. Four-year program trains residents in internal medicine and health disparities to work in resource poor settings.	Mentorship: · Most important program element identified by experienced providers · Mentor experienced provider in field of health disparities Didactic Teaching: · Training in systems necessary to make community-based programs function · Acquire clinical skills to work with wide range of problems in resource-poor settings · Skills in advocacy, leadership and operational management · Knowledge of public health, ethics, epidemiology, and medical anthropology · Language fluency Field Experiences: · Conduct quantitative or qualitative research on health disparities and global health · Spend time in World Health Organization understanding health policy Ambulatory continuity clinic in United State and continuity care clinic in resource-poor setting
Elit et al., 2011	Assessment of ethical issues and perspectives of Canadian medical students after experiences in international health electives (IHE). None had formal relationships between university and IHE.	Pre-departure Training: · Ethics surrounding clinical care, reciprocity, equity and solidarity · Consideration for participation motives and development of specific and realistic learning objectives · Learn about local setting including social, political and cultural context Supervision: · Supervision both at university and in local setting · Work to balance or reverse drain on local resources Structured debriefing after IHE completion
Provenzano et al., 2010	Ethical issues presented that occur from conducting short-term research projects in low resource settings by US based medical schools.	Develop Longstanding Partnership: · Comprehensive partnership ensures experience is meaningful and ethical through ongoing, equitable commitment to mutual opportunities · Fair and appropriate compensation for hosting research advisor, interpreters and logistical support to limit entrenching disparities · Ensure relevance to local community · Shared responsibility for project and capacity building Pre-departure Preparation: · Those selected should show performance, experience, motivation and personal character · Include cultural sensitivity and introductory language training · Case based training on research ethics and IRB approval Consultation with local stakeholders to develop project plans
Scarlett et al., 2011	Lessons learned over past decade from global health practice in Jamaica through a cross-disciplinary training program with University of the West Indies and other US partner universities.	Strong Institutional Synergies: · Multidisciplinary approach and multicultural emphasis · Mutually beneficial program based on previous institutional relationships Emphasis on Competency: · Promote scientific and cultural competencies · Promote critical thinking, problem-solving in resource-constrained settings and cultural competence · Provide orientation and discussion session prior to course Team-based Work: · Diversity of field visits and team work provided as part of course · Integration with international students Strong links with communities and cooperative learning environment
Redwood-Campbell et al., 2011	Steps involved in developing curriculum framework for global health in family medicine training programs by Ontario Family Medicine.	Identify Values and Principles: · Use of consensus to develop a mission statement · Core values and principles provide background context for competencies Develop Competencies: · Use curriculum, mentorship, practice opportunities and evaluation to develop knowledge and skills · Include theoretical and practical learning experiences Competencies include knowledge, skills and attitudes surrounding various areas of expertise required
Crump & Sugarman, 2010	Guidelines for ethics and training experiences developed by the Working Group on Ethics Guidelines for Global Health Training (WEIGHT).	Well-structured programs: · Sustained series of communication to delineate roles and responsibilities · Mutual and equitable benefits · Meet local needs and priorities through long-term partnerships · Promote transparency and safety · Recognize true costs Establish effective supervision: · Supervision and mentorship by host and sending institution · Facilitate communication Appropriate Trainees: · Formal training for trainees and mentors · Trainees should be adaptable, motivated, willing to listen and learn, and have abilities and experiences that match expectations · Stress recognition and respect for divergent views and cultural competency Knowledge of international guidelines

**Table 4:** Recommendation themes for training

Author/Year	In-Country Training	Long Term Commitment	Increase Impact	Mentorship	Preparation of Students
Leonard, 2002	x				
Spiegel et al., 2004		x	x		
Furin et al., 2006			x	x	x
Elit et al., 2011				x	x
Provenzano et al., 2010		x			x
Scarlett et al., 2011		x	x		x
Redwood-Campbell et al., 2011			x		x
Crump & Sugarman, 2010		x		x	x

Eleven articles were reviewed regarding effective strategies for clinical care (Table 5). Five articles were based on academic-national partnerships (Babich, Bicknell, Culpepper, & Jack, 2008; Quinn, 2008; Curioso et al., 2010; Crump, & Sugarman, 2008; Suchdev et al., 2007), four were based on NGO-national partnerships (Pfeiffer, 2003; Van den Broucke et al., 2009), and two were based on a local service location (Green et al., 2009; Cheah et al., 2010; Vidyasager, 2009; Sarriot et al., 2004). Nine of the eleven articles commented on collaborations/partnerships, seven commented on sustainability/capacity, seven commented on the meeting local needs, and five commented on process details such as training, evaluation or providing resources (Table 6).

**Table 5:** Summary of articles focused on clinical care

Author/Year	Context/Summary	Recommendations
Babich et al., 2008	Institutional commitment between Boston University and Lesotho to address human capital implications of HIV/AIDS. Assistance to the community focused on maximizing existing systems and resources, building workforce capacity and strengthening district level primary care services.	Mutual Partnership: · Begin with small targeted interventions to build relationship · Visible and widespread presence · Collaboration, negotiation, effective teams, and commitment to regular and complete communication · University can strengthen service mission and enrich teaching Clear Strategy Based on Needs: · Explore problems and needs · Strengthen both clinical and management aspects in coordinated manner · Remain flexible as priorities change or new problems arise · Ensure projects are complementary, not redundant Consider Sustainability: · Consider resources that are actually available and level of administration that can be maintained · Substantial initial investment and initial strategy to secure longer term funding
Quinn, 2008	Elaboration of initiatives and challenges experienced in establishing Johns Hopkins Center for Global Health as a way to facilitate and coordinate various international activities.	Central Coordinating Body: · Facilitate interdisciplinary and interdivisional collaboration · Recognize synergies and establish new relationships · Garner support from leadership · Integrate communication Develop Capacity: · Promote educational programs, symposia, forums and policy initiatives · Develop distance education to build societal capacity · Provide technical assistance and education to foreign professionals Provide Resources: · Provide access to catalog of existing programs and resources · Provide support through field training grants, scholarships, extramural funding
Pfeiffer, 2002	Ethnographic case study conducted in Mozambique discusses the impact of international aid on primary health care and the need for new collaboration between NGO aid workers and their local counterparts is discussed.	Equitable Relationships: · Industry wide international code of conduct for NGO activity regarding accountability and respect · Trusting and respectful professional relationships · Eliminate opportunities for NGOs to provide wide range of favors · Sensitize workers to impact of their presence and train how to engage positively in local community life Sustainability: · Technical assistance priorities determined by Ministries of Health rather than specific projects · Longer project cycles to ensure continuity · Build capacity through on-the-job training focus on transfer and routinization of skills rather than off-the-job seminars · Shift from focus on short-term results to long-term professional relationship Appropriate Evaluation: · Project evaluations focused on productive relationships, resulting transfer of skills rather than short term output Re-evaluations conducted 12months after project end
Curioso et al., 2010	Case report of international collaborative training program in biomedical informatics between University of Washington and Peru.	Collaboration: · Strong and sustained leadership facilitated start-up · Combination of short-term and long-term strategies directed at both individuals and institutions Tailored to Local Needs: · Accommodating local needs led to additional funding support · Promote re-entry grants to encourage researchers to return to home country
Van den Broucke et al., 2010	International collaboration project to strengthen the capacity for health promotion in South Africa.	Strengthen Capacity: · Use existing infrastructures · Create a broad-based network · Encourage communities to develop self reliance · Develop exit strategy to ensure sustainability Active Participation of Community: · Encourage participation in planning, implementation and evaluation · Use situation analysis to identify priority health issues, target groups and action strategies specific to area · Consultation with local stakeholders to develop project plans Collaboration: · Collaboration at both the international, and provincial to local level
Cheah et al., 2010	Experience and lessons learned from community engagement on the Thai-Burmese border.	Engage Community Help: · Ensuring advisory board is representative of population improves operational and ethical aspects of studies · One way to achieving compliance with international regulations creatively and practically · Need trust and respect of community Provide Training: Provide basic training sessions to allow effective engagement
Crump & Sugarman, 2008	Ethical considerations for short-term education and service initiatives experiences in global health programs.	Mutual and Reciprocal Benefit: · Benefits to institution should not trump responsibility to ensure program is beneficial to stakeholders · Balance tourism experiences with service experiences appropriately · Assess true costs of programs including direct and indirect, monetary and social · Consider if short term experiences are best use of resources Preparation of Trainees: · Consider time needed to orient trainees · Provide formal ethical guidance
Green et al., 2009	Exploratory study examining perceptions of utility and impact of short term medical missions trips in Guatemala.	Coordination with Local Providers: · Coordination reflects acknowledgement of competence that is perceived by local patient populations · Short term efforts are only effective if attached to a long-term program or attached to long term presence · Continuity of care can be provided when coordinated with long-term presence · The power and wealth differential makes trust, understanding and true partnership difficult, so active coordination is necessary Match Needs of Community: · Necessary to engage local healthcare community to determine what is needed · Poorly aligned projects remove or lessen incentive of government to invest in healthcare in the country Minimize Burden and Maximize Impact: · Limit team to only those necessary and use country personnel as possible · Donate equipment, medications and supplies Focus on populations most in need
Suchdev et al., 2007	Principles for sustainable short-term international medical trips based on experiences of University of Washington and rural community in El Salvador.	Target Efforts Appropriately: · Refer to mission regularly to ensure projects stay on track · Educate teams, community and peers to understand context and facilitate understanding · Commit to work the community needs and wants · Determine needs of population and gather adequate supplies that use existing infrastructure for ongoing care Teamwork: · Assist host country in taking ownership of their community health issues · Supervise team members in manner consistent with policies of patient care in developed country · Involve team members with diverse expertise Sustainability: · Work in a single location that can be augmented with successive trips · Demonstrate commitment to ongoing relationship · Work within existing systems of care and employ local physicians · Conduct periodic evaluation and gather feedback to ensure meeting outcomes and developing interventions appropriately
Vidyasagar., 2009	Discussion of various models for technology transfer to developing countries based on experiences of American International Health Alliance	Develop Partnerships: · Consider national impact value and ‘knowledge footprint’ · Identify needs and develop memorandums of understanding to establish rules of engagement and lines of communication · Develop core group of leaders and ‘train the trainers’ · Ensure orientation to regional history and culture and cultural sensitivity · Develop mutually agreeable plan and evaluate periodically Sustainability: · Enhance skills of caregivers and provide new tools · Need strong commitment to program at highest level of authority in host country · Develop strong infrastructure with appropriate budget · Plan for sustainability from the start
Sarriot, et al., 2004	Framework and process to map progress towards sustainable child health to systematically approach dimensions of evaluation on progress for sustainability assessment.	Capacity of Local Organization and Community: · Efforts at capacity building should be judged in parallel to progress achieved · Create dialogue around goals, roles and strategies in order to select objectives that the local system considers important · Enable communities to select and test appropriate indicators · Help stakeholders take ownership by envisioning their future after the project Sustainability: · Necessary to consider health outcomes, health services, organizational capacity and viability, community competence/capacity, and larger context · Use evaluation of sustainability to design better programs and policies in future · Plan for sustainability from beginning of project

**Table 6:** Recommendation themes for clinical care

Author/Year	Collaboration/Partnerships	Sustainability/Capacity	Meeting Local Needs	Process Comments
Babick et al., 2008	x	x	x	
Quinn, 2008	x	x		x
Pfeiffer, 2002	x	x		x
Curioso et al., 2010	x		x	
Van den Broucke et al., 2010	x	x	x	
Cheah et al., 2010			x	x
Crump & Sugarman, 2008	x			x
Green et al., 2009	x		x	x
Suchdev et al., 2007	x	x	x	
Vidyasagar, 2009	x	x		
Sarriot, et al., 2004		x	x	

## 4. Discussion

### 4.1 Effective Strategies for Research

Many of the articles reviewed focused on ethical considerations or frameworks for research, rather than on a specific global health research initiative, however, six of the eleven noted important process considerations when conducting research projects. First, the need for appropriate funding to allow for all phases of the research and capacity development in the host country was discussed (Treloar & Graham, 2003; Tugwell et al., 2006). Treloar and Graham (2003) noted the importance of standardization when conducting qualitative research due to the inexperience of most researchers with the methodology (Treloar & Graham, 2003). Lack of standardization, particularly when working with a multidisciplinary approach, can lead to inconsistency in implementation as well diminishing motivation in process utilization (Treloar & Graham, 2003). However, it was also noted that research projects need to allow for flexibility to ensure investigators can respond to needs and concerns, specifically of the community (Treloar & Graham, 2003; Simon et al., 2007). Simon et al. (2007) found that keeping a flexible design to allow for input and cooperation of the community can help address ethical concerns of conducting research on vulnerable populations (Simon et al., 2007). Providing appropriate amounts of information to ensure research goals are clear, and informed consent is truly informed is also important (Lavery et al., 2010; Macrae, 2007). Additionally, it was found that development of a community advisory board promotes more effective communication and enables dissemination of research goals while also addressing community needs (Simon et al., 2007). Lastly, Nishtar (2004) discussed the importance of transparency for appropriate development of policies and procedural frameworks based on the research (Nishtar, 2004), noting that third party accountability has a key role in the effective utilization of global policies and frameworks for effective research procedures (Nishtar, 2004).

The second recommendation for global health research was consideration of the social, political, and economic context. Mosavel et al. (2005) noted that the research context is important to consider in order to work alongside policy, rather than in opposition to it (Mosavel et al., 2005). It also assists in addressing the tension between research and service delivery that can lead to ethical challenges (Mosavel et al., 2005). Recognition of the complexity of the situation provides exploration of possible pathways to impact the research concern, rather than simply addressing one problem (Huynen et al., 2005). Additionally, providing for continued discussion when developing research plans, recognizes the importance of respect and collaboration with the community, and develops a research process, rather than a single project focus (Simon et al., 2007; Tugwell et al., 2006). This further enables cultivation of a collaborative forum wherein both researcher and host country are given a voice to shape real community needs while also generating knowledge to address health outcomes (Simon et al., 2007). Techniques such as dissemination into national or regional journals, rather than only international journals provides for more policy and practice impact in the host country, and integration of research into health programs develops capacity of the community and host institution (Costello & Zumla, 2000; Tugwell et al., 2006). Recognizing that the research should benefit society rather than the partners and posing minimal risk to vulnerable populations by taking into account expected therapeutic results are also important aspects of considering the context upfront to make the most impact (Nishtar, 2004; Macrae, 2007).

Capacity of the host country was continually mentioned in articles regarding effective global health research strategies, however, not all defined capacity in the same manner. A portion of the articles focused on capacity of the research partners in the host country, and the importance of collaboration and interdisciplinary work. Rojas et al. made a case for promoting development and sustainability of capacity by using available resources in the host nation, discussing designs and ethical issues, and assigning the bulk of funding to the resource-poor country (Rojas et al., 2007). Costello and Zumla (2000) also noted the importance of line management of funds in the local institution, as well as providing advice and technical support (Costello & Zumla, 2000). It was recommended to promote the collaborative process by assigning specific roles to each partner and holding regular meetings to gather feedback (Rojas et al., 2007; Nishtar, 2004). The necessity of drawing from multiple fields, such as medicine, epidemiology, sociology, political science, education and economics, was also suggested as a way to get the best research product, while also building partnerships with the host country (Huynen et al., 2005). Nurturing partnerships and building trust through shared decision making, joint priority setting, and developing national research networks was recommended (Costello & Zumla, 2000; Tan-Torres, 1999).

The last recommendation was building capacity in the host country through engaging the community and strengthening relationships with stakeholders. Mosavel et al. (2005) recommended inviting local critique and feedback, and involving non-mainstream community members to accomplish this end Mosavel et al., 2005). Providing opportunities for community questions and training community members to help conduct the research were regular suggestions to build rapport and trust (Simon et al., 2007; Mosavel et al., 2005; Lavery et al., 2010; Tan-Torres, 1999). Additionally, engaging community members and utilizing collaborative processes unveiled underlying determinants of poor health while in turn empowering community members to further engage in health promotion (Simon et al., 2007). Strategic selection of sites, seeking to understand perceptions rather than just measure them, and working to respect dissenting opinions in the community were noted by Lavery et al. (2010) to both build capacity and establish relationships (Lavery et al., 2010). Lastly, increasing translation and impact through appropriate knowledge sharing, and packaging of results was necessary for developing capacity in low and middle income countries to develop equity research (Tugwell et al., 2006).

### 4.2 Effective Strategies for Training

Most articles categorized as addressing training were focused on the students and training program, with only one article focused on training within the host country. Using a comparison between NGOs and government health care systems, Leonard suggested systems should allow flexibility and invest in organizational quality (Leonard, 2002). Based on the recommendations, training of staff should result in increasing the reputation of the government health care system. The importance of completeness of examinations, appropriate use of laboratory tests, and attentiveness to the client and health education should be addressed in training for staff (Leonard, 2002).

The importance of institutional commitment and long-term partnerships were discussed in four of the articles (Scarlett et al., 2011; Spiegel et al., 2011; Provenzano et al., 2010; Crump, & Sugarman, 2010). Institutional commitment was recommended as necessary for success and sustainability of initiatives, allowing for continuity and establishment of networks (Spiegel et al., 2011). It was also recommended to ensure experiences are meaningful, ethical, and relevant to the local community (Spiegel et al., 2011; Provenzano et al., 2010; Crump, & Sugarman, 2010). Scarlet et al suggested using previous institutional relationships to build a mutually beneficial program (Scarlett et al., 2011). The Working Group on Ethics Guidelines for Global Health Training (WEIGHT) recommends well-structured programs in order to delineate roles and responsibilities, ensure benefits are mutual, and promote transparency and safety (Crump & Sugarman, 2010). Recognition of true costs and fair and appropriate compensation are both noted as important to this process (Provenzano et al., 2010; Crump, & Sugarman, 2010).

Four articles commented on specific aspects of the program used to increase impact. Two commented on the importance of a clear vision (Spiegel et al., 2011; Redwood-Campbell et al., 2011) and two noted the use of field experiences (Scarlett et al., 2011; Furin et al., 2006). Spiegel et al. (2011) emphasized the importance of a vision to keep the focus on application of knowledge and allow for evaluation of intended outcomes (Spiegel et al., 2011). Core values and principles can also be used as a background context for competencies when developing a curriculum (Redwood-Campbell et al., 2011). Furin et al. (2006) developed strategic field experiences to increase the impact of didactic teaching provided in a resident training program (Furin et al., 2006). Similarly, Scarlett et al. (2011) used a diversity of field visits and teamwork to teach team-based work and the importance of a cooperative learning environment (Scarlett et al., 2011).

Mentorship was noted as an important aspect in three articles. Furin et al. (2006) stated that mentorship was the most important program element (Furin et al., 2006). It was suggested to establish mentorship at both the university and local setting in order to balance or reverse the drain on local resources commonly seen in short term trips (Elit et al., 2011; Crump, & Sugarman, 2008). Mentorship would also provide for structured debriefing and facilitate communication (Elit et al., 2011; Crump, & Sugarman, 2008).

Preparation of students was the most commonly mentioned recommendation in the articles reviewed. Specific topics to include in pre-departure training varied, but some topics were consistent including ethics, cultural competency, and language fluency (Furin et al., 2006; Elit et al., 2011; Provenzano et al., 2010; Crump, & Sugarman 2010). In addition to building a knowledge base, articles also noted the importance of teaching skills such as advocacy, operational management, critical thinking, and problem-solving (Scarlett et al., 2011; Furin et al., 2006; Redwood-Campbell et al., 2011). A number of articles also commented on the importance of selecting the best applicants by including consideration of a participant’s performance, motivation, experience, personal character, and willingness to listen and learn (Furin et al., 2006; Elit et al., 2011; Provenzano et al., 2010; Crump, & Sugarman, 2010).

### 4.3 Effective Strategies for Clinical Care

Articles categorized as addressing clinical care commented on four major topics: collaboration/partnerships, sustainability/capacity, meeting local needs, and process specific recommendations. The vast majority of the articles commented on the need for mutually beneficial partnerships involving commitment to collaboration and equitable relationships. Authors noted the importance of trusting and respectful professional relationships (Green et al., 2009; Pfeiffer, 2009), strong and sustained leadership (Green et al., 2009; Quinn, 2008; Curioso et al., 2010), and the need to ensure programs were beneficial to both institutions and stakeholders (Babich et al., 2008; Crump, & Sugarman, 2008; Vidyasager, 2009). While each article noted recommendations to accomplish this in specific settings, most projects would benefit from improved communication (Babich et al., 2008; Quinn, 2008; Vidyasager, 2009), and development of partnerships across various levels (Van den Broucke et al., 2009). When considering on-the-ground efforts, Babich et al. (2008) noted the importance of a visible and widespread presence through small initial interventions in order to build relationships (Babich et el., 2008). On the university side, Quinn noted the need for a central coordinating body to recognize synergies and establish new relationships (Quinn, 2008). Pfeiffer noted the need to add coordination as a core criterion for assessment of projects, rather than focusing on short-term output evaluation (Pfeiffer, 2003). Green commented on the importance of coordinating with local providers to acknowledge competence and improve continuity of care (Green et al., 2009). Suchdev et al. (2007) and Vidyasager (2009) noted the importance of partnership with the community (Suchdeve et al., 2007; Vidyasager, 2009). It was also noted that consideration of national impact and evaluation of mutually developed plans assist in developing partnerships (Vidyasager, 2009).

A second major theme noted in the articles focused on clinical care was the need for a focus on sustainability of the project through capacity building at the local level. Sustainability recommendations ranged from up-front project considerations such as assessing resources that are actually available (Babich et al., 2008), to end-of-the-project considerations such as exit strategies (Van den Broucke, 2009). A number of different types of capacity building activities were noted such as educational programs, symposia, and on-the-job training (Quinn, 2008; Pfeiffer, 2003). Pfeiffer (2003) pointed out the importance of building capacity through development of productive, long-term relationships and a transfer of skills, rather than simply providing workshops that may take workers away from their crucial duties (Pfeiffer, 2003). Vidyasagar (2009) also discussed the importance of sustainability when transferring skills and noted the importance of commitment both from high levels of authority and community stakeholders (Vidyasager, 2009). The use of existing infrastructure and focus on long-term impact were noted as promoting sustainability in projects (Babich et al., 2008; Pfeiffer, 2003; Van den Broucke et al., 2009; Vidyasager, 2009), and periodic evaluation was suggested to ensure programs are meeting outcomes and developing interventions appropriately (Suchdev et al., 2007; Sarriot et al., 2004). Sarriot et al. (2004) developed a framework to evaluate sustainability, including considerations of health outcomes, health services, organizational capacity and viability, community competence/capacity, and the larger environmental context (Sarriot et al., 2004).

The necessity of considering local needs was the third theme noted in the articles on clinical care. Exploring problems and needs of the community/country, accommodating local needs in the overarching project goals, and encouraging participation in all aspects of the project were noted as effective methods (Babich et al., 2008; Curioso et al., 2010; Van den Broucke et al., 2009). Cheah et al. (2010) noted the need for trust and respect of the community and the importance of considering representativeness of the feedback gathered (Cheah et al., 2010). Alignment with local needs also helps avoid situations where the government decreases investment in healthcare based on counts of programs, without attention to quality or longevity (Green et al., 2010). Van den Broucke et al. (2009) used the specific method of situation analysis to identify priority health issues, target groups and action strategies. Other articles stated the importance of this process for success, but didn’t state a specific method used (Van den Broucke et al., 2009).

Finally, five of the articles gave recommendations specific to processes such as evaluation, training and providing resources. Quinn (2008) noted at the university level, the importance of providing resources such as access to existing programs and support through funding (Quinn, 2008). Pfeiffer (2003) suggested conducting re-evaluations 12 months after the project end to measure the long-term impact and encourage consideration of sustainability (Pfeiffer, 2003). Cheah et al. (2010) and Crump and Sugarman (2008) both noted the importance of training, both for local stakeholders and visiting clinicians, to ensure an appropriate knowledge base and appreciation of ethical considerations (Cheah et al., 2010; Crump, & Sugarman, 2008). Limitation of team personnel, donation of equipment, and focus on populations most in need are also processes that can minimize the burden and maximize the impact of programs (Green et al., 2009).

## 5. Conclusions

This review provides evidence on effective strategies in conducting global health research, training, and clinical care by identifying the common structures used in implementation of programs as well as the key elements that allowed for program success, and the common barriers experienced. In defining global health as the “health problems, issues, and concerns that transcend national boundaries and [that] may be addressed by cooperative actions” (Institute of Medicine, 1997), three main implications for the development of new global health programs can be seen in this review. The first is the need for new programs to be developed within the framework of formal long-term arrangements. A number of articles noted that better coordination resulted from long-term partnerships. Long-standing commitments also help ensure experiences are mutually beneficial, meaningful and ethical. Using small projects to build trust and develop relationships was suggested as an effective process. While formal partnerships can be time-consuming and require effort to develop, the commitment can increase impact and sustainability. The second main implication of this review is the necessity of engaging local partners from the onset of program development. Focusing on local needs and understanding of local political, social and cultural conditions leads to more ethical research, prepared trainees, and effective clinical care. Exploration of problems and needs through consultation with stakeholders assists in ensuring programs are truly accomplishing their mission and vision. The third is regular recommendations to evaluate programs in order to determine if goals are being met, programs are needs based and sustainable, and the best outcomes are being reached. Considerations of sustainability, partnership building, and organizational capacity were mentioned as part of evaluation topics, in addition to the more common endpoints of outcomes and access.

There are several limitations to this review. First selected articles were limited to publications in the English language between 2001 and 2011, which could have led to exclusion of important articles in other languages and prior to 2001. Second, since studies with positive results are more likely to be published, the studies in this review may represent successful programs with positive program evaluation results. Lastly, the difference in methodology used by each article limited the amount of comparison that could be made between articles. So a formal meta-analysis could not be performed and would not have been appropriate. Therefore, conclusions from this review are qualitative and meant to guide future program development.

Evidence from this review suggests that global health program development should be completed within the framework of a larger institutional commitment or partnership. Support from leadership in the university or NGO, and an engaged local community are both integral to success and sustainability of efforts. It is also important for program development to engage local partners from the onset, jointly exploring issues and developing goals and objectives. Evaluation is a recommended way to determine if these goals are being met, and should include considerations of sustainability, partnership building, and capacity. Global health research programs should consider details regarding the research process, the context of research, partnerships, and community relationships. Training for global health should involve mentorship, pre-departure preparation of students, and elements developed to increase impact. Clinical care programs should focus on collaboration, sustainability, meeting local needs, and appropriate process considerations.
